# Posttraumatic stress disorder predicts poor health-related quality of life in cardiac patients in Palestine

**DOI:** 10.1371/journal.pone.0255077

**Published:** 2021-07-27

**Authors:** Hala Allabadi, Abdulsalam Alkaiyat, Tamer Zahdeh, Alaa Assadi, Aya Ghanayim, Shaden Hasan, Dalia Abu Al Haj, Liana Allabadi, Salim Haj-Yahia, Christian Schindler, Marek Kwiatkowski, Elisabeth Zemp, Nicole Probst-Hensch

**Affiliations:** 1 Department of Epidemiology and Public Health, Swiss Tropical and Public Health Institute, Basel, Switzerland; 2 University of Basel, Basel, Switzerland; 3 Faculty of Medicine and Health Sciences, An-Najah National University, Nablus, Palestine; 4 Faculty of Graduate Studies, Arab American University, Ramallah, Palestine; 5 School of Clinical Sciences, University of Bristol, Bristol, United Kingdom; 6 Institute of Cardiovascular and Medical Sciences, Glasgow University, 126 University Place, Glasgow, United Kingdom; University of Belgrade, SERBIA

## Abstract

**Background:**

The longitudinal association of posttraumatic stress disorder (PTSD) with health-related quality of life (HRQL) in cardiac patients’ remains poorly studied, particularly in conflict-affected settings.

**Materials and methods:**

For this cohort study, we used baseline and one-year follow-up data collected from patients 30 to 80 years old consecutively admitted with a cardiac diagnosis to four major hospitals in Nablus, Palestine. All subjects were screened for PTSD and HRQL using the PTSD Checklist Specific and the HeartQoL questionnaire. We used a generalized structural equation model (GSEM) to examine the independent predictive association of PTSD at baseline with HRQL at follow-up. We also examined the mediating roles of depression, anxiety, and stress at baseline.

**Results:**

The prevalence of moderate-to-high PTSD symptoms among 1022 patients at baseline was 27∙0%. Patients with PTSD symptoms reported an approximate 20∙0% lower HRQL at follow-up. The PTSD and HRQL relationship was largely mediated by depressive and anxiety symptoms. It was not materially altered by adjustment for socio-demographic, clinical, and lifestyle factors.

**Discussion:**

Our findings suggest that individuals with a combination of PTSD and depression, or anxiety are potentially faced with poor HRQL as a longer-term outcome of their cardiac disease. In Palestine, psychological disorders are often stigmatized; however, integration of mental health care with cardiac care may offer an entry door for addressing psychological problems in the population. Further studies need to assess the effective mental health interventions for improving quality of life in cardiac patients.

## Introduction

Cardiovascular diseases (CVDs) account for 31% of all global deaths [1]. The Middle East has a high burden of CVD morbidity, mortality, and premature cardiac events [2], leading to a substantial proportion of patients experiencing disability [3]. Subsequently, there is a strong need for cardiac rehabilitation with a special focus on improving health-related quality of life (HRQL). There is evidence that HRQL predicts adverse health outcomes including mortality and hospitalizations in cardiac patients [4].

Targets for intervention in cardiac rehabilitation include psychosocial stressors and mental health disorders, which tremendously impact overall HRQL. Depression in particular is not only an established risk factor for CVD [5], but is increasingly recognized for its adverse prognostic effect in cardiac patients [6]. Less is known about the prognostic role of posttraumatic stress disorder (PTSD), a common psychiatric disorder in individuals who experience a traumatic event (e.g. war, natural disasters, physical assault, life threating illness) [7].

Numerous cross-sectional studies have found CVD and its risk factors are more prevalent among individuals with PTSD than those without PTSD [8]. This higher CVD prevalence can reflect both the etiological role of PTSD and the traumatic effect of a cardiac event. On the one hand, a recent meta-analysis has shown that PTSD is associated with a 55% increased incidence of cardiac disease or cardiac-related mortality, even after adjustment for demographic, clinical, and psychosocial factors including depression [9]. On the other hand, CVD events can be psychologically traumatic experiences given their sudden onset, unpredictable consequences and life-threatening nature: approximately 10∙0–30∙0% of people suffer from PTSD after a cardiac event such as a myocardial infarction (MI) or cardiac arrest [10–12].

Yet, evidence on whether and how the presence of PTSD in cardiac patients predicts the course of their disease and quality of life remains limited. The few existing studies suggest that PTSD in cardiac patients is associated with impaired psychosocial functioning, poor physical health [13], decreased HRQL [14], non-adherence to medication and treatment [11], as well as an increased risk of mortality, morbidity and recurrent cardiac events [15]. These adverse associations of PTSD with HRQL may be partly mediated by other mental health symptoms (e.g. depression, anxiety or stress), also associated with heart disease patients’ HRQL [16, 17].

To the best of our knowledge, the longer-term impact of PTSD on HRQL among cardiac patients has not been studied in the occupied Palestinian territories (oPt). The present study among a well-characterized cohort of cardiac patients in the Palestinian population a) investigates the independent predictive association of PTSD with HRQL over a one-year follow-up period, and b) assesses the mediating role of depression, anxiety and stress.

## Materials and methods

### Study design and sample

**Fig 1:**
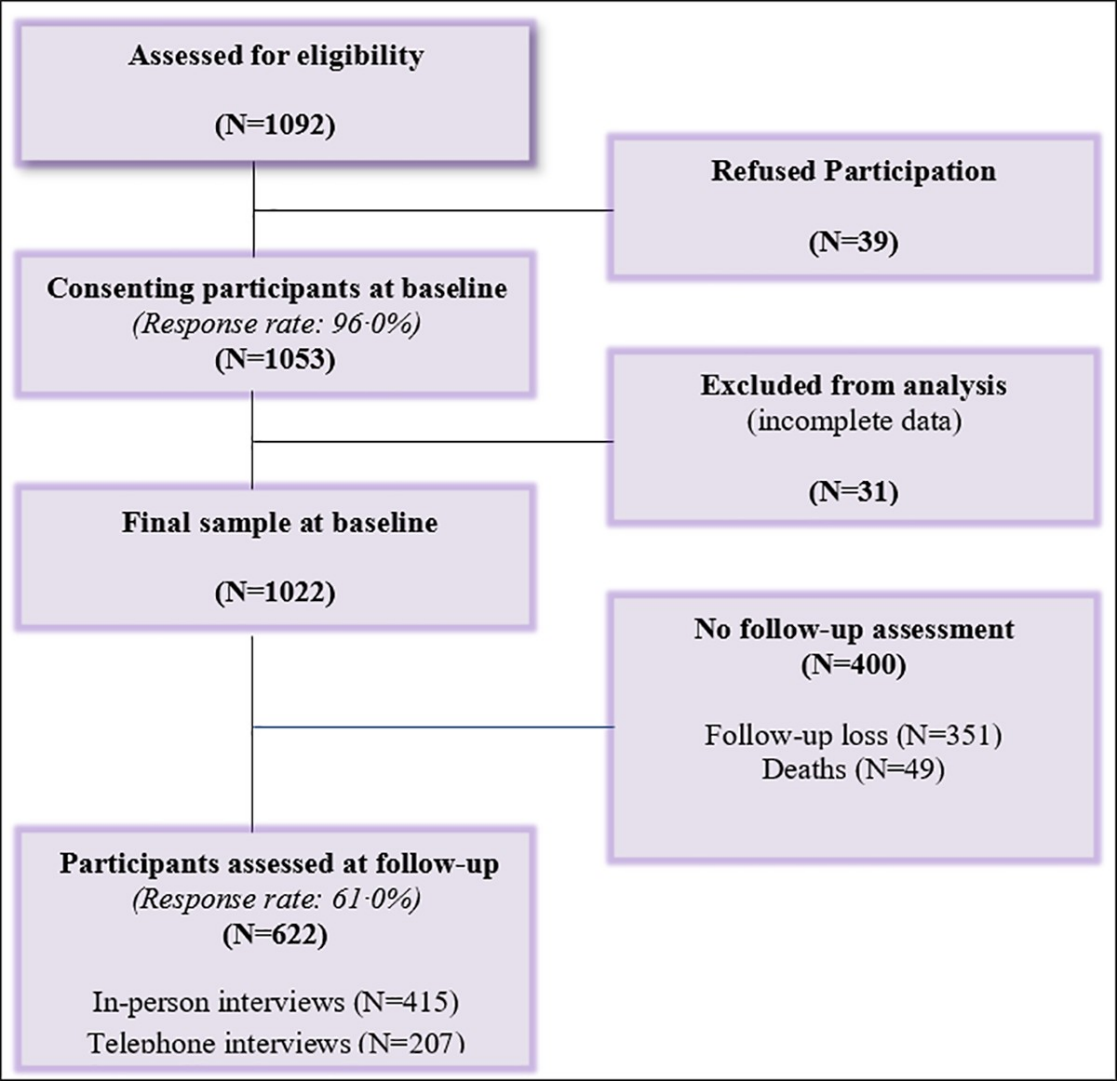
Flowchart from baseline recruitment to longitudinal study sample.

A longitudinal design was used to explore the predictive association between PTSD and HRQL in hospitalized cardiac patients after a 12-month follow-up. This study followed cardiac patients consecutively admitted to cardiology units at four major hospitals in Nablus, Palestine. The recruitment and procedures of the study and data from the baseline study have been described in detail elsewhere [18]. To be eligible for the study, patients were required to be between 30 to 80 years old and have clinically diagnosed CHD, MI, angina, heart failure, or any other cardiac disease. Patients were recruited for the initial baseline assessment within one week of hospitalization (range 1–7 days) between March, 2017 and November, 2017. With a participation rate of 96∙0%, a total of 1053 patients were included in the study. Thirty four patients were excluded from the baseline analysis and follow-up due to incomplete data. A follow-up assessment was conducted approximately one year after enrollment in the study between June, 2018 and December, 2018 (Fig 1). Ethical approval for the study was obtained from the Ethics Committee of Nordwest-und Zentral Schweiz (EKNZ) (Project ID: 2017–00235) in Basel, Switzerland, and the Institutional Review Board (IRB) committee at An-Najah National University in Nablus, Palestine. All participants provided a written informed consent at baseline and follow-up.

### Procedures

Trained medical students from An-Najah University carried out in-person interviews using a structured questionnaire containing validated assessment tools. At follow-up, all assessed patients who provided consent at baseline were re-contacted by telephone and invited to attend an in-person interview at a medical facility in Nablus, Palestine. Patients refusing in-person interviews were asked to provide relevant information during a phone interview. In this current study, PTSD, depression, anxiety, stress, socio-demographic, clinical, psychosocial and lifestyle factors obtained from all participants at baseline assessment were the independent variables of interest. HRQL was assessed as the study outcome at 12-month follow-up. An overview of the assessment tools has been described in detail previously [18].

### Assessments

#### Predictor variable: Posttraumatic stress disorder (PTSD)

At baseline, PTSD was assessed by the PTSD Checklist Specific (PCL-S), a 17-item questionnaire corresponding to PTSD symptoms based on the Diagnostic and Statistical Manual of Mental Disorders, 4th Edition (DSM-IV) criteria. Each item in the questionnaire is scored from 1 (not at all) to 5 (extremely), indicating the extent to which the patient has been bothered by the symptom during the previous month. A total PCL-S score is determined by summing up the scores of all items (range 17–85). In the present study, the total score was divided into two categories of PTSD symptom severity: “no-to-little symptoms” (PCL-S scores 17–29) and “moderate-to-high symptoms” (PCL-S scores ≥30; cutoff for clinically relevant PTSD) [19]. In the current sample, the PCL-S demonstrated an internal consistency (Cronbach α) of 0∙86 for all 17 items.

#### Mediating variables: Depression, anxiety and stress

At baseline, depression, anxiety and stress were measured using the Depression Anxiety Stress Scale-42 (DASS-42) by Lovibond & Lovibond [20]. The questionnaire is divided into three scales (depression, anxiety and stress), with items scored on a four-point Likert scale ranging from 0 (did not apply to me at all) to 3 (applied to me very much) measuring the extent to which each state was experienced over the past week. Scores for each of the three scales are determined by summing up the scores and categorized into: depression 0–9 (normal), 10–20 (mild-moderate), ≥21 (severe-very severe); anxiety 0–7 (normal), 8–14 (mild-moderate), ≥15 (severe-very severe); stress 0–14 (normal); 15–25 (mild-moderate), ≥26 (severe-very severe) [20]. Cronbach α for the three scales in our sample was 0∙92 (DASS-depression), 0∙82 (DASS-anxiety), and 0∙89 (DASS-stress), respectively.

#### Outcome variable: Health-related quality of life (HRQL)

At follow-up, HRQL was assessed using the HeartQoL questionnaire, a 14-item ischemic heart disease (IHD)-specific scale which measures overall quality of life. The global HeartQoL score indicates how the patient perceives being bothered by their heart disease [21]. In this study the total score was determined by summing up the scores of all 14 items which were assessed on a four-point scale from 0 to 3 (0 = poor and 3 = good quality of life). The overall score range was 0–42, with higher scores indicating better HRQL. In the present study, Cronbach’s α for HRQL was 0∙91.

#### Other variables of interest

*Socio-demographics factors*. Age, gender, marital status, education, employment and residence were assessed by interview at baseline.

*Clinical factors*. Clinical data related to the cardiac disease including diagnosis, treatment procedure, comorbidities, duration of disease, medications at discharge and somatic symptoms, which were assessed using the Patient Health Questionnaire-15 (PHQ-15), were retrieved from medical records and by interview at baseline.

*Lifestyle factors*. Information on smoking status, body-mass index (weight (kg)/height (m)^2^), and physical activity (e.g. the number of days of physical activity for at least 30 minutes per week) were obtained through in-person interviews at baseline.

#### Statistical methods

**Fig 2:**
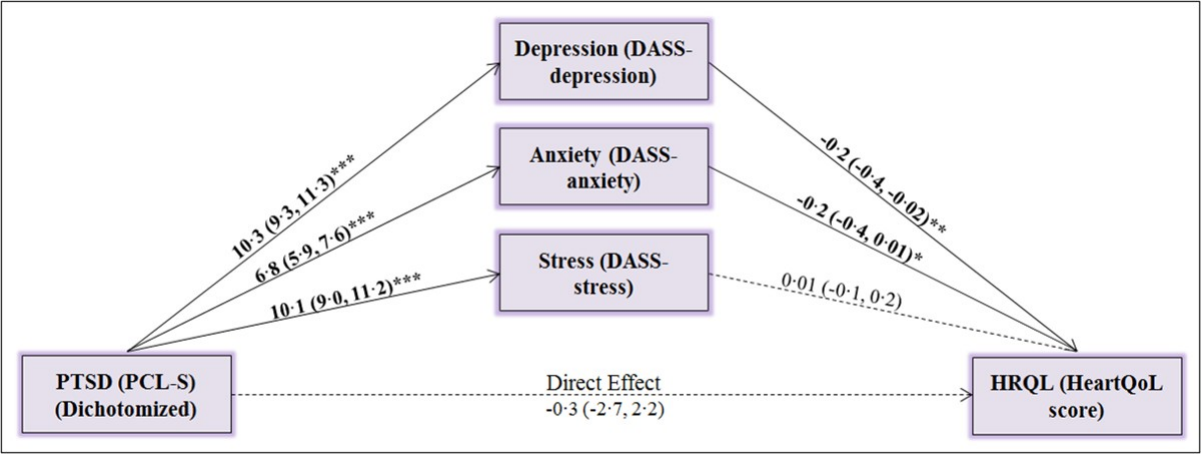
GSEM: The estimation of the direct and indirect effects of PTSD on HRQL. Covariates additionally adjusted for: Age, gender, occupation, education, residence, hospital, cardiac diagnosis, cardiac treatment, years with disease, somatic symptoms (PHQ-15), comorbidities, medications, body mass index, physical activity, smoking status. Follow-up duration was also adjusted for with no differences in the results. Bolded lines indicate statistically significant effects and dashed lines indicate non-significant effects. Each line is labeled with the respective effect estimate and its 95%-confidence intervals. Note that because the scales of the three mediating variables are not the same, the effect sizes cannot be compared directly. Follow-up for HRQL: (n = 622). PTSD = Post-Traumatic Stress Disorder, PCL-S = Post-Traumatic Stress Disorder Checklist, DASS = Depression, Anxiety, Stress Scale, HRQL = Health-related quality of life, Questionnaire. *p<0∙10, **p<0∙05, ***p<0∙001.

PTSD symptoms were analyzed as the dichotomous variable (presence/absence of moderate-high PTSD symptoms). To assess the relationship between PTSD at baseline and HRQL at follow-up involving potential mediators obtained at baseline, we built a generalized structural equation model (GSEM), portrayed in Fig 2. The model is based on the hypothesis that depression, anxiety and stress mediate the relationship between PTSD and HRQL. The analysis was adjusted for age, gender, occupation, education, residence, hospital, cardiac diagnosis, cardiac treatment, years with disease, somatic symptoms (PHQ-15), comorbidities, medications, body-mass index, physical activity, smoking status and duration of follow-up. We selected these covariates a priori, and based on evidence for association with PTSD as likely confounders of the association between PTSD and HRQL. All GSEM models were estimated using maximum likelihood in the STATA Statistical Software Release 15 (StataCorp., College Station, U.S.A.).

## Results

### Recruitment

Of the 1022 participants included at baseline, n = 622 (61∙0%) were reexamined at follow-up. The mean duration of the approximate one-year follow-up was 399 (SD = 93∙7) days (range: 235–677 days). Among the patients whom we attempted to contact, 415 (40∙6%) attended in-person interviews and 207 (20∙2%) were interviewed by phone; while 351 patients (34∙4%) were lost to follow-up (not reached after four telephone calls; phones disconnected). Telephone interviews were conducted with patients who were not able to attend in-person interviews due to (1) physical restrictions, (2) lack of means of transportation, or (3) no interest in attending the appointment. For deceased people (n = 49, 4∙8%), a telephone interview with the next of kin was used to ascertain or confirm the cause and date of death.

### Sample characteristics at baseline

At baseline, among the 1022 patients, the mean age was 58∙9±10∙1 years (range 30–80 years). Of these participants, 73∙4% were male, 90∙6% were married, 37∙0% were unemployed, and 58∙7% did not have a high school diploma. With regard to clinical variables, 39∙7% had an acute MI, 32.7% had established CHD, 15∙9% had stable or unstable angina, and 11∙7% had some other type of cardiac disease, including mitral or aortic valve stenosis, valve regurgitation, heart block and others. Among the category of other diagnoses, 29 (2∙8%) patients had heart failure. There were no major socio-demographic or lifestyle differences at baseline among participants included vs excluded in the follow-up assessment. However, depression, complicated treatments, and number of medications were slightly more common among non-participants at follow-up (S1 Table).

### Distribution of socio-demographic, clinical, psychosocial and lifestyle characteristics according to PTSD status

The mean scores of PTSD symptoms at baseline and follow-up were 27∙3 (SD = 9∙5; range 17–71), and 23∙5 (SD = 11∙0; range 17–80), respectively and the PTSD prevalence’s were 27∙0% and 21∙2%, respectively. Characteristics of participants by PTSD status at baseline are shown in Table 1. Among our sample with PTSD symptoms, there were more females (39∙0% vs 23∙2%); there were more unmarried individuals (43∙7% vs 25∙7%); housewives [40∙4% vs 24∙6% (professional occupation status)]; individuals with severe cardiac diseases [36∙7% vs 17∙5% (angina)]; individuals diseased over 10 years [33∙1% vs 24∙6% (less than one year)]; patients who had a CABG [31∙8% vs stent (23∙9%)]; had two or more comorbidities [32∙6% vs 20∙8% (no comorbidities)]; and more individuals who scored high on the PHQ-15 [41∙1% vs 13∙0% (low somatic symptoms)]. Additionally, among those with PTSD symptoms, patients were more likely to be depressed [53∙9% vs 10∙6%]; stressed [39∙7% vs 5∙7%]; and anxious [38∙5% vs 10∙6%]; more likely to be never-smokers [34∙3% vs 22∙1% (current smoker)]; and physically inactive [34∙3% vs 22∙4% (daily)]. None of the patients confirmed when asked at baseline that in the context of their cardiac care they had ever been asked about their mental health well-being.

**Table 1:** Socio-demographic, clinical, psychosocial and lifestyle characteristics at baseline, by PTSD status, *n* = 1022.

	Moderate-to-high PTSD symptoms (PCL-S ≥30) *(n = 279)*	No-to-low PTSD symptoms (PCL-S < 30) *(n = 743)*
***Socio-demographic factors***		
Age	58∙8±10∙3	59∙1±10∙1
Gender		
Female	106 (39∙0)	166 (61∙0)
Male	173 (23∙2)	571 (76∙8)
Marital status		
Married	237 (25∙7)	689 (74∙3)
Not married	42 (43∙7)	54 (56∙3)
Residence		
City	115 (24∙4)	356 (75∙6)
Village	137 (29∙0)	339 (71∙0)
Camp	48 (64∙0)	27 (36∙0)
Education degree		
No high school diploma	180 (30∙0)	420 (70∙0)
High school diploma	64 (24∙5)	197 (75∙5)
College degree	35 (22∙2)	126 (77∙8)
Occupation		
Professional	51 (24∙6)	156 (75∙4)
Non-professional	62 (20∙2)	245 (79∙8)
Unemployed	125 (33∙1)	256 (66∙9)
Retired	22 (27∙5)	58 (72∙5)
House wife	19 (40∙4)	28 (59∙6)
***Clinical Factors***		
Cardiac diagnosis		
Coronary heart disease	116 (34∙7)	218 (65∙3)
Myocardial infarction	91 (22∙5)	317 (77∙5)
Angina	28 (17∙5)	132 (82∙5)
Other	44 (36∙7)	76 (63∙3)
Years with cardiac disease		
Less than one year	154 (24∙6)	472 (75∙4)
Two-nine years	79 (31∙1)	178 (68∙9)
10 or more years	46 (33∙1)	93 (66∙9)
Cardiac treatment (at admission)		
Catheterization/stent	127 (23∙9)	405 (76∙1)
Catheterization /coronary bypass artery graft	76 (31∙8)	166 (68∙2)
Catheterization/other & unknown	76 (30∙6)	172 (69∙4)
Co-morbidities		
None	62 (20∙8)	236 (79∙2)
One	80 (26∙6)	224 (73∙4)
Two or more	137 (32∙6)	283 (67∙4)
Medications		
None	33 (25∙0)	99 (75∙0)
One to two	39 (25∙8)	112 (74∙2)
Three to four	207 (28∙1)	532 (71∙9)
***Psychosocial factors***		
Depression		
Depressed (DASS-Depression ≥ 10)	213 (53∙9)	185 (46∙1)
Not depressed (DASS-Depression ≤ 9)	66 (10∙6)	558 (89∙4)
Anxiety		
Anxiety (DASS-anxiety ≥8)	236 (38∙5)	380 (61∙5)
No Anxiety (DASS-anxiety ≤7)	43 (10∙6)	363 (89∙4)
Stress		
Stress (DASS-stress ≥15)	258 (39∙7)	395 (60∙3)
No stress (DASS-stress ≤14)	21 (5∙7)	348 (94∙3)
***Lifestyle factors***		
Smoking status		
Never	125 (34∙3)	239 (65∙7)
Former	47 (27∙6)	123 (72∙4)
Current	107 (22∙1)	381 (77∙9)
Physical activity		
None	115 (34∙3)	223 (65∙7)
Not daily	53 (28∙0)	136 (72∙0)
Daily	111 (22∙4)	384 (77∙6)
Body-mass index		
Normal weight	56 (27∙4)	148 (72∙6)
Overweight	111 (25∙7)	321 (74∙3)
Obese	112 (29∙2)	274 (72∙8)

Data are mean (SD) or n (%). PTSD = Posttraumatic stress disorder, PCL-S = Posttraumatic stress disorder Checklist Specific, PHQ = Patient Health Questionnaire, DASS = Depression, Anxiety, Stress Scale. Physical activity defined as the number of days of physical activity for at least 30 minutes per week.

### Mediation analyses: Direct and indirect associations of PTSD with HRQL

The mean HRQL score at follow-up was 28∙2 (SD = 12∙1). The mean HRQL score among those with PTSD at baseline was 24∙6 (SD = 12∙8) compared to 30∙0 (SD = 11∙4) among those without PTSD (p <0∙001).

Results from the mediation analyses (Fig 2) report the associations of PTSD with each potential mediating factor (depression, anxiety, stress) and between each potential mediator and HRQL, after adjusting for covariates. PTSD was a positive predictor of depression (β = 10∙3, p<0∙001), anxiety (β = 6∙8, p<0∙001) and stress (β = 10∙1, p<0∙001). In turn, depression (β = -0∙2, p = 0∙025) and anxiety (β = -0∙2, p<0∙068), but not stress predicted HRQL at follow-up, implying depression and anxiety to be mediators for the relationship between PTSD and HRQL. Furthermore, the direct effect of PTSD on HRQL was not statistically significant after controlling for covariates and mediators (β = -0∙3, p = 0∙824), indicating the predictive association of PTSD with HRQL at one year follow-up was largely mediated by depression and anxiety. Our reference model further identified treating hospital, duration of disease, comorbidities and physical activity as independent predictors of HRQL. These factors neither modified the association of PTSD with depression or anxiety, nor the association of depression and anxiety with PTSD.

## Discussion

This is the first study on the role of PTSD on the quality of life of cardiac patients in Palestine. Although there was some improvement in PTSD symptoms at follow-up, patients with moderate and high PTSD symptoms reported lower HRQL, approximately one year after baseline assessment. The PTSD and HRQL relationship was largely mediated by depressive and anxiety symptoms. The negative effect of PTSD remained evident after adjusting for relevant covariates. The careful control for confounding and the longitudinal nature of our study strengthens the belief that this relationship may be causal.

Previous studies in non-cardiac patients have shown that greater PTSD severity was associated with poorer psychosocial and physical HRQL, with a decline in HRQL similar in size, as in the current study [22–24]. There has been extensive research on the relationship between PTSD and HRQL among war veterans and those with various chronic illnesses, but little on the relationship of PTSD and HRQL, specifically among CVD patients. Our results are consistent with the few similar studies of CHD patients, which found PTSD symptoms are associated with a lower quality of life [14, 25]. A further study which investigated HRQL among patients with PTSD following cardiac arrest reported more problems in overall quality of life and decreased HRQL scores compared to those without PTSD [26]. Decreases in HRQL among cardiac patients with PTSD compared to those without PTSD were of similar magnitude as observed in the current study. The 20% decrease in HRQL observed in this study is expected to increase re-hospitalizations and mortality, as low HRQL has been found to be an independent and strong predictor of life expectancy and hospitalizations in patients with different heart diseases [27].

To our knowledge, this is the first study to assess the specific mediating role of depression, anxiety and stress in the relationship between PTSD and HRQL among cardiac patients. Our findings are similar to those of a previous study conducted among a small sample of motor vehicle accident survivors, in which an indirect effect of PTSD on quality of life through depression and anxiety was also found [28]. Numerous studies have also found that approximately half of people with PTSD had major depressive disorder (MDD) across different sub-samples [29, 30], which is consistent with our analyses. Moreover, a body of literature has documented the independent associations of depression and anxiety with poor HRQL among CVD patients [16, 17].

Anxiety and depression, possibly resulting in part from a high prevalence of PTSD in conflicted Palestine, have previously been found to influence post-operative recovery and cardiac-specific quality of life after open heart surgery [31]. Deterioration of HRQL post-intervention in patients with heart disease depends on factors directly related to the course of disease (e.g. surgical complications, progression of non-cardiac diseases, or neurological and psychological complications after cardiopulmonary bypass). However, presence of anxiety and depression either as result of the diagnosis or pre-existing before disease onset can additionally worsen HRQL not only the mental health aspect, but could also influence the course of the disease by modifying susceptibility to aspects such as pain or poor physical functioning [32]. Anxiety and depression may thereby influence HRQL by impacting on unhealthy lifestyles (e.g. smoking, alcohol consumption, physical inactivity, obesity), health seeking behavior or treatment adherence [33]. Furthermore the adverse effect of anxiety and depression on HRQL was also found to mediate in part the adverse effect of type D personality on HRQL in heart disease patients [31].

The prevalence of PTSD in our sample of cardiac patients was 27∙0%, which is considerably higher than the PTSD rates reported in previous studies showing associations between PTSD and quality of life in different groups of patients. Cohen et al., who assessed for PTSD and HRQL among CHD patients found a prevalence of 9∙0% [14]. The prevalence of PSTD in our study exceeds PTSD rates found among patients with trauma [34], stroke [35], and hematological malignancies [36].

One of the major strengths of the present study is its longitudinal design, which allowed studying the predictive association of PTSD with HRQL in a clear temporal resolution. The detailed characterization of the study population limited residual confounding in studying the independent association of PTSD with HRQL. Moreover, previous research has focused on only one category of cardiac patients, rather than considering a sample of diverse cardiac diagnoses. Further strengths of this study are the high participation rate and the use of the HeartQoL questionnaire, which is specifically designed to assess quality of life in cardiac patients.

The findings in this present study should be interpreted taking into consideration several limitations. First, we cannot exclude the possibility that depression, anxiety and stress symptoms measured among those with PTSD pre-dated the traumatic event itself in some patients. Second, the baseline assessment was conducted shortly after admission following a cardiac event, when elevated stress and anxiety are to be expected, thus our estimates of the magnitude of effect may not be generalizable to a longer follow-up. Third, we did not assess HRQL at baseline. If HRQL at follow-up was correlated with HRQL at baseline, the association observed would suggest an association of PTSD with the persistence of decreased HRQL. Finally, individuals who died before the follow-up assessment, refused to participate in the follow-up, or could not be reached could in fact have had a very low quality of life. If they were also more likely to exhibit PTSD symptoms, we may have underestimated the effect of PTSD on HRQL.

## Conclusion

In the conflict-affected area of Palestine, high rates of PTSD as well as depression and anxiety are observed among cardiac patients [18]. The current results suggest that individuals with a combination of PTSD and depression or anxiety are potentially faced with poor HRQL as a longer term outcome of their heart disease. Despite general recommendations for the integration of mental health care into cardiac rehabilitation, none of the patients in this study reported having been assessed for psychological problems or asked about their mental health. In Middle East settings such as Palestine, psychological disorders are often stigmatized, however integration of mental health care with cardiac care may offer a unique entry door for efficiently addressing psychological problems in a considerable part of the population. CVDs affect a high percentage of the population. Patients and their family members may be particularly receptive for mental health interventions in a period of life where faced with disability and death. Further studies need to assess the culturally acceptable and effective mental health interventions for improving quality of life in heart disease patients. This is particularly pressing given the recent aggression of political conflict in Palestine on top of the COVID-19 pandemic [37]. The combination of traumatic factors likely leads to a further increase in the prevalence of PTSD and thus a further decrease in HRQL of cardiac patients, not only in the Palestinian setting but also as a result of the impact of the COVID-19 pandemic, globally.

## Supporting information

S1 Table Sample characteristics among patients included vs excluded at follow-up, n = 1022. Data are mean (SD) or n (%). PTSD = Posttraumatic stress disorder, PCL-S = Posttraumatic stress disorder Checklist Specific, PHQ = Patient Health Questionnaire, DASS = Depression, Anxiety, Stress Scale. ^*†*^ = Independent *t*-test. Physical activity defined as the number of days of physical activity for at least 30 minutes per week; *P* values in bold are significant at p <0∙05.
(PDF)
