# Supplementary material for: Posttraumatic stress disorder predicts poor health-related quality of life in cardiac patients in Palestine
Source: PLoS One. 2021 Jul 27;16(7):e0255077. doi: 10.1371/journal.pone.0255077 (PMC8315523; doi:10.1371/journal.pone.0255077)
Supplement: S1 Table — Data are mean (SD) or n (%). PTSD = Posttraumatic stress disorder, PCL-S = Posttraumatic stress disorder Checklist Specific, PHQ = Patient Health Questionnaire, DASS = Depression, Anxiety, Stress Scale. † = Independent t-test. Physical activity defined as the number of days of physical activity for at least 30 minutes per week; P values in bold are significant at p <0∙05. (PDF) [file pone.0255077.s001.pdf]

S1 Table. Sample characteristics among patients included vs excluded at follow-up, n=1022

	**Included at follow-up (n=622)**	**Excluded at follow-up (n=400)**	***P* value**
Socio-demographic factors			
Age	58·1±10·1	60·3±10·0	<0·001
Gender			
Female	152 (24·6)	120 (30·0)	
Male	470 (75·4)	280 (70·0)	**0·055**
Marital status			
Married	566 (91·4)	357 (89·2)	
Not married	56 (8·6)	43 (10·8)	0·243
Residence			
City	288 (46·5)	183 (45·7)	
Village	292 (46·7)	184 (46·0)	
Camp	42 (6·8)	33 (8·3)	0·682
Education degree			
No high school diploma	356 (57·5)	241 (60·2)	
High school diploma	169 (26·8)	97 (24·3)	
College degree	97 (15·7)	62 (15·5)	0·626
Occupation			
Professional	125 (20·2)	84 (21·0)	
Non-professional	206 (32·8)	103 (25·7)	
Unemployed	209 (33·8)	167 (41·8)	
Retired	51 (8·2)	30 (7·5)	
House wife	31 (5·0)	16 (4·0)	0·061
Clinical Factors			
Cardiac diagnosis			
Coronary heart disease	197 (31·8)	136 (34·0)	
Myocardial infarction	249 (40·2)	156 (39·0)	
Angina	104 (16·3)	60 (15·0)	
Other	72 (11·7)	48 (12·0)	0·864
Years with cardiac disease			
Less than one year	388 (62·7)	239 (59·7)	
Two-nine years	144 (22·8)	113 (28·3)	
10 or more years	90 (14·5)	48 (12·0)	0·110
Cardiac treatment (at admission)			
Catheterization/stent	340 (54·9)	192 (48·0)	
Catheterization/coronary artery bypass graft	149 (24·1)	91 (22·7)	
Catheterization/other & unknown	133 (21·0)	117 (29·3)	**0·010**
Co-morbidities			
None	193 (31·2)	105 (26·2)	
One	183 (29·5)	119 (29·8)	
Two or more	246 (39·3)	176 (44·0)	0·187
Medications			
None	94 (14·7)	40 (10·0)	
One-two	86 (13·9)	66 (16·5)	
Three-four	442 (71·4)	294 (73·5)	0·066
Somatic symptoms (PHQ-15)			
Minimal (PHQ ≤ 4)	52 (8·4)	38 (9·5)	
Low (PHQ 5-9)	148 (23·9)	92 (23·0)	
Medium (PHQ 10-14)	209 (33·3)	108 (27·0)	
High (PHQ ≥ 15)	213 (34·4)	162 (40·5)	0·113
Psychosocial factors			
PTSD (PTSD-PCL-S)			
Medium-high PTSD symptoms (PTSD ≥30)	197 (31·8)	119 (29·8)	

No-low PTSD symptoms (PTSD <30)	425 (68·2)	281 (70·2)	0·484
Depression			
Depressed (DASS-Depression ≥ 10)	229 (36·5)	169 (42·2)	
Not depressed (DASS-Depression ≤ 9)	393 (63·5)	231 (57·8)	0·066
Anxiety			
Anxiety (DASS-anxiety ≥8)	363 (58·2)	252 (63·0)	
No Anxiety (DASS-anxiety ≤7)	259 (41·8)	148 (37·0)	0·123
Stress			
Stress (DASS-stress ≥15)	395 (63·3)	259 (64·7)	
No stress (DASS-stress ≤14)	227 (36·7)	141 (35·3)	0·644
Lifestyle factors			
Smoking			
Never	217 (34·6)	149 (37·2)	
Former	100 (16.1)	70 (17·5)	
Current	305 (49·3)	181 (45·3)	0·454
Physical activity			
None	194 (30·9)	145 (36·2)	
Not daily	114 (18·4)	74 (18·5)	
Daily	314 (50·7)	181 (45·3)	0·162
Body-mass index			
Normal weight	124 (19·5)	82 (20·5)	
Overweight	263 (42·5)	167 (41·7)	
Obese	235 (38·0)	151 (37·8)	0·933

Data are mean (SD) or n (%). PTSD= Posttraumatic stress disorder, PCL-S= Posttraumatic stress disorder Checklist Specific, PHQ=Patient Health Questionnaire, DASS=Depression, Anxiety, Stress Scale. [†]=Independent *t*-test. Physical activity defined as the number of days of physical activity for at least 30 minutes per week; *P* values in bold are significant at p <0·05
